# Children’s learning-by-teaching with a social robot *versus* a younger child: Comparing interactions and tutoring styles

**DOI:** 10.3389/frobt.2022.875704

**Published:** 2022-10-31

**Authors:** Lena Pareto, Sara Ekström, Sofia Serholt

**Affiliations:** ^1^ Division of Media and Design, School of Business, Economics and IT, University West, Trollhättan, Sweden; ^2^ Department of Education, Communication and Learning, University of Gothenburg, Gothenburg, Sweden; ^3^ Division of Learning, Communication and IT, Department of Applied IT, University of Gothenburg, Gothenburg, Sweden

**Keywords:** peer tutoring, learning-by-teaching, robot tutee, video analysis, comparative study, robot *versus* human, child–robot interaction, mathematics game

## Abstract

Human peer tutoring is known to be effective for learning, and social robots are currently being explored for robot-assisted peer tutoring. In peer tutoring, not only the tutee but also the tutor benefit from the activity. Exploiting the learning-by-teaching mechanism, robots as tutees can be a promising approach for tutor learning. This study compares robots and humans by examining children’s learning-by-teaching with a social robot and younger children, respectively. The study comprised a small-scale field experiment in a Swedish primary school, following a within-subject design. Ten sixth-grade students (age 12–13) assigned as tutors conducted two 30 min peer tutoring sessions each, one with a robot tutee and one with a third-grade student (age 9–10) as the tutee. The tutoring task consisted of teaching the tutee to play a two-player educational game designed to promote conceptual understanding and mathematical thinking. The tutoring sessions were video recorded, and verbal actions were transcribed and extended with crucial game actions and user gestures, to explore differences in interaction patterns between the two conditions. An extension to the classical initiation–response–feedback framework for classroom interactions, the IRFCE tutoring framework, was modified and used as an analytic lens. Actors, tutoring actions, and teaching interactions were examined and coded as they unfolded in the respective child–robot and child–child interactions during the sessions. Significant differences between the robot tutee and child tutee conditions regarding action frequencies and characteristics were found, concerning tutee initiatives, tutee questions, tutor explanations, tutee involvement, and evaluation feedback. We have identified ample opportunities for the tutor to learn from teaching in both conditions, for different reasons. The child tutee condition provided opportunities to engage in explanations to the tutee, experience smooth collaboration, and gain motivation through social responsibility for the younger child. The robot tutee condition provided opportunities to answer challenging questions from the tutee, receive plenty of feedback, and communicate using mathematical language. Hence, both conditions provide good learning opportunities for a tutor, but in different ways.

## 1 Introduction

Tutoring can be described as when “people who are not professional teachers are helping and supporting the learning of others in an interactive, purposeful, and systematic way” (Topping, 2000, p. 6). Substantial evidence indicates that human tutoring is effective, where both participants gain an understanding (Bloom, 1984; Topping, 2000; Chi et al., 2001; Topping et al., 2003; Roscoe & Chi, 2007). One-to-one tutoring is the most exclusive form of a teaching–learning situation, comprising one teacher and one learner. The teaching–learning activity can be tailored to the participants with respect to knowledge levels, preferences, and needs. Peer tutoring, where the tutor and tutee have similar age and knowledge levels (Topping, 2000), is a way to organize learning in education. It is a method of cooperative learning where students are organized in pairs, assigned the roles of tutor and tutee, and given a common goal in a pre-planned teaching–learning situation (Duran & Monereo, 2005). Typically, the tutor has more advanced domain knowledge than the tutee, but the knowledge gap can be minimal (Roscoe & Chi, 2007).

Peer tutoring has shown learning gains for tutors and tutees in primary school mathematics, as justified by several previous meta-reviews where peer tutoring was found to be superior to traditional solitary math-book learning (Alegre et al., 2019). The authors argue that peer tutoring is an effective method for mathematics since it encourages participation and empowers cooperative and inclusive learning. Accordingly, this has inspired the development of technology-assisted learning-by-teaching scenarios using, e.g., teachable agents (cf., Biswas et al., 2001; Schwartz et al., 2007). Taking on the role to teach someone else stimulates the tutor to engage in activities such as explaining and answering tutee questions, which are beneficial for their learning.

Humanoid robots have been used in research to explore the potential of robot-assisted peer tutoring in education. In a recent review of robot studies in the classroom (Woo et al., 2021), the robot’s role as a peer was the most common and occurred in 6 of 23 studies (26%). Chen et al. (2020) compared giving a robot the roles of tutor, peer, and tutee in relation to a collaborative game. They argued for giving the robot a peer-like role, where the robot expresses tutee and tutor behaviors. In a review of social robots’ potential in education, Belpaeme et al. (2018) concluded that robot-assisted peer tutoring has shown learning outcomes similar to that of human tutoring, in restricted, well-defined tasks. They argue that the physical presence of the robot substantially contributes to these results and makes social robots favorable to traditional learning technologies in a tutoring context. Duran & Monereo (2005) also highlighted the importance of a well-structured situation and a shared objective to achieve productive peer tutoring—features that a game can provide.

Studies on robot tutees are still scarce and comprised only 9% of the studies examined by Belpaeme et al. (2018). Since then, designing the robot as a peer (of which tutee is one type) has gained interest.

In this study, we explore a learning-by-teaching peer tutoring situation where primary school children act as tutors to both a robot tutee and a child tutee while playing a two-player collaborative mathematics game. The robot-augmented version of the game (Pareto et al., 2019) was co-designed with teachers and students (Barendregt et al., 2020). The current tutee resulted from developments based on the co-design process, stakeholder recommendations, and findings from a previous study focusing on interaction trouble and children’s repair strategies (Serholt et al., 2020).

To our knowledge, this is the first study of its kind seeking to directly compare a robot tutee with credible tutees such as younger children. The child tutees are children a few years younger than the tutors. The tutoring task consists of teaching both the robot tutee and the child tutee to play the game. In a parallel article, we studied the tutees’ subjective perceptions of the tutoring sessions (Serholt et al., 2022) and found that the tutors considered communication and collaboration with the younger child to be significantly easier than with the robot. They also stated that they needed more help from the teacher when working with the robot and what they were expected to do during the session was not as clear when working with the robot. However, the two situations were comparable with respect to the tutors’ enjoyment and willingness to engage with either tutee again and their assessments of learning gains for both tutees and themselves.

### 1.1 Research aim

The aim of this study is to explore how a learning-by-teaching situation with a robot tutee differs concerning tutoring actions and teaching styles, from an equivalent situation with a younger child. The aim is to reveal mechanisms providing learning-by-teaching opportunities for the tutor in the two situations, and thereby discuss the benefits and drawbacks of both approaches. To this end, we conduct video analysis and adopt an explorative approach, taking inspiration from previous tutoring studies involving humans only. The following research question guided this study:

RQ: how do children’s tutoring sessions with a robot tutee compare to tutoring sessions with younger children with respect to tutoring actions and teaching styles?

## 2 Background

In this section, we present literature on peer tutoring and learning-by-teaching. Then, we describe research on robots as tutees. Finally, we present the IRFCE framework for analyzing educational discourse structures, which has informed our research approach.

### 2.1 Peer tutoring

Tutoring is an old practice, originating in ancient Greece (Topping, 2000). Tutoring has other benefits than teaching for the learner and the teacher. Tutors do not necessarily need to be experts in the subject domain; rather, it is considered favorable if the tutor is just slightly more knowledgeable than the tutee (Topping, 2000; Roscoe & Chi, 2007; Alegre et al., 2019). Students can be better tutors than adults since they have more recently learned the material and, therefore, relate more naturally to the problems a tutee may face (Duran & Monereo, 2005). Moreover, peer tutors are more likely to share the same linguistic styles, vocabulary, and other preferences. Even young children have learned to tutor effectively (Topping, 2000).

Topping (2000) compared peer tutoring and professional teaching and listed potential benefits of peer tutoring as more guided practice, individualization, and variation; more questioning, modeling, and demonstrations; more prompting and self-correction; more feedback and praise; and more ownership of the learning process including meta-cognition and self-regulation. The tutor and the tutee benefit. Topping (2000) also listed potential risks with peer tutoring: overall poor support, misleading or incorrect guidance, lack of error detection or misconceptions, and impatience to finish the task. The author points out the importance of well-structured tutoring tasks, and that tutoring effects rely on the quality of the actual implementation. Games are considered suitable candidates for well-structured tutoring tasks (Duran & Monereo, 2005) and can provide meaningful shared tutoring activities of underlying mathematical concepts without explicit training (Topping et al., 2003).

A study of peer tutoring scenarios where children had to explain and justify their ideas and solutions to each other in a cooperative mathematical game showed that verbalizations were beneficial for the children to reflect on their own understanding (Topping et al., 2003). The study also found that peer tutoring increased self-esteem for tutors and tutees and that their tutoring sessions increased in quantity and quality regarding the tutoring discussions about mathematics, with an evident gain for both tutors and tutees (Topping et al., 2003). According to a meta-review of 51 studies of peer tutoring in primary school mathematics education, cross-age tutoring had better results than same-age tutoring (Alegre et al., 2019). The by far most analyzed variable regarding peer tutoring was academic achievement, often with positive results, but the authors argue that other variables such as motivation, attitude, and mathematical self-concept can be addressed by peer tutoring as well.

### 2.2 Tutor learning: mechanisms of learning-by-teaching

In a literature review on learning-by-teaching, Duran (2017) claimed that learning-by-teaching was first demonstrated in peer tutoring studies in the early 1960s, where it was found that tutors learned even more than the tutees. This captured the interest of researchers seeking to unpack the mechanisms involved in learning-by-teaching. In the examined studies, a relationship between the activity and tutor learning could be deciphered: more complex teaching also yields more learning opportunities (Duran, 2017).

Peer tutors can benefit from tutoring by engaging in reflective knowledge building through interactive communication while teaching someone (Roscoe & Chi, 2007). Teaching includes activities such as planning how to explain and describe the material, which can stimulate self-explanation (Koh et al., 2018). Tutors can benefit from preparation activities by reviewing their understanding and reorganizing their knowledge. Another common activity in teaching is questioning (Duran, 2017). Tutors can ask questions to guide the tutee and evaluate their progression, but tutors will also have the responsibility to answer questions from their tutee. Hence, learning opportunities can arise from trying to respond to unexpected questions from the tutee or from handling the tutee’s confusion to inadequate, incomplete, or contradictory explanations or actions (Roscoe & Chi, 2007). Here, the tutee functions as a reflective mirror of the tutor’s teaching, which can reveal knowledge deficits in the tutor’s understanding and introduce new ideas. These knowledge-building activities establish grounds for the learning-by-teaching paradigm (Duran, 2017).

However, the learning effectiveness of peer tutoring depends on the quality of the tutor–tutee interactions, e.g., the quality of the tutor’s explanations, the tutee’s responses, and provided feedback (Roscoe and Chi, 2007). Tutoring provides opportunities to be involved in reflective knowledge-building such as producing quality explanations, self-reflecting, or evaluating one’s knowledge to identify weak spots (Roscoe and Chi, 2007). Motivation to engage comes from the so-called protégé-effect, meaning that the tutor feels responsible for the tutee’s learning, shown to motivate children to invest more time when teaching an agent than when learning for themselves (Chase et al., 2009). However, tutors do not always take advantage of such opportunities; many tutors limit themselves to a uni-directional approach (or “knowledge telling”) (Duran, 2017). Moreover, Martino and Maher (1999) showed that timely and challenging questions fostered an understanding of primary school mathematics, meaning that tutors can benefit from tutee questions. Yet, the quality of questions is a key factor for reflective knowledge-building (Duran, 2017), and asking good questions is difficult even for adults (King 1994).

To summarize, learning-by-teaching requires mutual engagement and a bi-directional interaction where the tutor and tutee both engage in an activity and the tutee creates challenges for the tutor to be effective (Duran, 2017). Teaching that is uni-directional, on the other hand, provides limited opportunities for the tutor to learn (Duran, 2017). Hence, peer tutoring needs to be carefully designed. For a child–child situation, the main concerns are the tutoring activity and the matching of peers; for a robot–child situation, it is a design challenge. There is a growing interest in designing technology-assisted support for peer tutoring activities, which for the learning-by-teaching scenario means designing a tutee that stimulates tutor learning. For this purpose, humanoid robots as tutees are promising alternatives (Jamet et al., 2018). For all the aforementioned reasons, it is interesting to examine which tutoring actions evolve in learning-by-teaching situations with child tutees compared to robot tutees.

### 2.3 Peer tutoring with robots

There has been growing interest in the area of peer tutoring with social robots in recent years. For instance, a care-receiving robot tutee was studied by Tanaka & Matsuzoe (2012), where young children taught the robot to speak English through spontaneous caregiving actions. Significant learning effects were reported for the tutors. Furthermore, when a child tutor instructed a robot tutee on how to write through corrective feedback, promising learning effects concerning motivation and the targeted skill were observed (Lemaignan et al., 2016). Similarly, in a study by Yadollahi et al. (2018), children taught a robot tutee reading skills and also through corrective feedback when the robot made mistakes. The tutoring activity turned out to be beneficial for high reading-ability students but distracting for the low reading-ability group. In a study by Chen et al. (2020), they instead studied the effectiveness of the robot’s role. The role was examined in three variants: as a pure tutor, a pure tutee, and a combination thereof (referred to as reciprocal peer learning). The domain was vocabulary learning, and they used an educational collaborative game for the tutoring activity. In the tutee condition, the robot lacked vocabulary knowledge, in the tutor condition, the robot made no mistakes and knew all of the words, and in the reciprocal peer condition, the robot altered between the two. The reciprocal peer robot condition showed more benefits regarding language learning and affect compared to the pure roles.

In terms of design, Jamet et al. (2018) described three main characteristics of a robot tutee based on their literature review of robots in learning-by-teaching scenarios, i.e., that the robot is programmed to make mistakes, that it develops some skills and thus appears to be learning, and that it engages in the teaching scenarios with verbal and gestural communication. The features of the tutors are less specified in the literature, but the authors argue that a didactic contract should be specified including the targeted goal, the role of the tutor, and the knowledge gap between the tutor and tutee.

### 2.4 Frameworks for studying educational discourse structures

A classical way to examine educational discourse structures and classroom interactions is through the teaching framework IRF (Sinclair, & Coulthard, 1975), referring to initiation (I), response (R), and feedback (F). It states that classroom interactions typically comprise three phases: *initiation*, when the teacher poses a question; *response*, when the student(s) reply to the question; and *feedback*, from the teacher to the student’s response. In peer tutoring situations, the IRF framework is too limited (Duran, 2017). The framework was extended by Graesser and Person (1994) to accommodate additional situations comprising richer structures such as tutoring, amounting to the IRFCE framework. The new phases refer to collaboration (C) and evaluation (E). In the extended structure, initiation (I) refers to when the tutor poses a question or states a problem, followed by a response (R) from the tutee and some feedback (F) from the tutor, but then the actual collaboration (C) takes place where the tutor and tutee engage in dialog trying to solve the problem or answer the original question. Finally, the tutor evaluates (E) the tutees’ understanding of their improved response. Duran and Monereo (2005) extended the framework further, by identifying another interaction pattern called ICE, typical for reciprocal peer tutoring. The ICE pattern begins with an initiation (I) from either part and then the tutor and the tutee engage in a cooperative (C) cycle in which their mutual response or solution is constructed through questions and hints. Finally, the tutor evaluates (E) the situation. It is to be noted that Duran and Monereo (2005) referred to the (C) phase as cooperation rather than collaboration since they distinguish between two types of cooperation both characterizing tutoring situations: tutoring and collaboration. Tutoring refers to cooperation with an asymmetrical role division, e.g., the tutor initiates actions and the tutee reacts, whereas collaboration refers to symmetric cooperation where initiatives and roles are reciprocal. Duran & Monereo (2005) found that the tutorial sequence (IRFCE) was more common in fixed-role tutoring situations, whereas the collaborative sequence (ICE) was more characteristic of reciprocal tutoring.

## 3 Materials and methods

**FIGURE 1:**
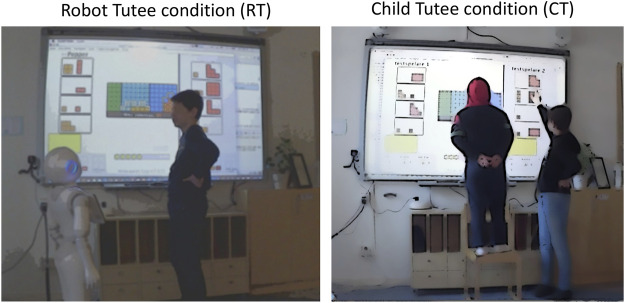
Still image from rear camera video capture of a tutoring session with the robot (left) and younger student (right).

As mentioned previously, this study is based on the same study as reported by Serholt et al. (2022), i.e., a small-scale field experiment carried out in a Swedish school. The study followed a within-subject design wherein students in the sixth grade (12–13-year-olds) acted as tutors for a third-grade student (9–10-year-olds) and a robot tutee in separate sessions (see Figure 1). The tutoring task consisted of playing a mathematical game targeting conceptual understanding and reasoning (Pareto, 2014), and the robot used was the Pepper robot from Softbank Robotics. Serholt et al. (2022) explored the tutors’ subjective perceptions of the two tutoring sessions based on a set of quantitative measures, whereas the current study explores differences in interaction patterns between the child and robot tutee through video analysis based on the extended IRFCE discourse structure framework (Duran & Monereo, 2005) described previously.

### 3.1 The START system

The apparatus used for this study was the Student Tutor and Robot Tutee (START) system (Pareto et al., 2019), where a social robot was designed and developed to function within an existing mathematics game. To this end, we have augmented a virtual teachable agent within the graphical arithmetic game with an embodied robot tutee. The arithmetic game with a virtual teachable agent, which was entirely text-based, has previously shown learning effects regarding motivation, conceptual understanding, and mathematical thinking (Pareto et al., 2011; Pareto et al., 2012; Pareto, 2014). The aim of the START system is to study the potential of learning-by-teaching with social robots.

**FIGURE 2:**
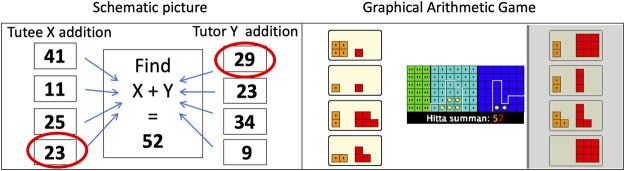
Mini-challenge from the game “Find the pair up to 100” that was used in the study; a schematic illustration (left) and a screenshot from within the graphical arithmetic game (right).

The physical setup of the START system comprises a wall-mounted interactive whiteboard displaying the game and the robot tutee. The robot is connected to the game through a local wireless network so that it can react to the tutor’s actions and respond based on the situation in the game. The game is a two-player card and board game with a series of mini-games of varying difficulty, ranging from “Ten-buddies” with the goal to reach the sum of 10 by combining two cards, to more advanced games involving division, multiplication, and negative numbers. For this study, we used a mini-game called “Find the Pair up to 100” comprising mini-challenges of finding a given sum by choosing one card from each player’s hand that equals the sum. A mini-challenge example is shown in Figure 2, where 52 is the displayed sum to be found with two cards, e.g., one from the left hand with cards 41, 11, 25, or 23, and one from the right hand with cards 29, 23, 34, or 9. There is always at least one matching pair in each mini-challenge, here, 23 + 29 = 52.

In the graphical arithmetic game used in the study (see Figure 2, right), all mathematical values are graphically represented by colored blocks instead of numbers. The blocks differ in color depending on if they represent values in tens (orange) or ones (red), such that, e.g., the top-left card in the graphical arithmetic game screenshot with four orange blocks and one red block represents the number 41. The players have a maximum of four cards each to choose from for every mini-challenge. Thus, there are normally 16 possible card combinations to consider for the sum. The players are encouraged to discuss and agree on which cards to choose since a laid card cannot be retracted. The players can engage in strategic discussions on how to be clever in finding the pair, e.g., by excluding all cards greater than the sum, by adding the red blocks (the ones) or orange blocks (the tens) first to exclude options, or by systematically going through all combinations in some order. The players have to engage in approximate or exact mental calculations to judge each proposed pair. Since the difficulty level of a mental integer addition depends on, e.g., integer size and the carry-over operation (Buijsman & Pantsar, 2020), the difficulty of the mini-challenges varies. Once a card is selected, the number, i.e., the colored blocks, is added to the common game board in the middle through an animation visualizing adding the number of blocks. It is then the other player’s turn to select a matching card, which is also added to the game board to complete the arithmetic addition. For each correct pair, a star is added to a scoreboard. If the sum of the chosen cards is not the displayed sum, a dialog box explaining the difference appears. The mini-game is finished when the players have completed 10 mini-challenges (i.e., expended their cards).

### 3.2 Robot tutee design

Taken together, the robot’s behavior includes gesturing, gaze, text-to-speech, and automatic speech recognition based on pre-programmed keywords in Swedish for verbal communication. The START system is fully autonomous such that there is no “Wizard of Oz” involved. A robot action is either triggered from the game (such as the game questions or comments when scoring), from a player’s verbal or physical interactions with the robot (such as responses, comments, or touching the robot’s hand), or after a while of silence. The robot is designed to be active, engaged, and inquisitive and is programmed to ask questions related to the current situation in the gameplay. The type of question the robot asks in a given situation is determined by the game and depends on the tutee’s current knowledge level according to a zone of proximal development schema previously used for the teachable agent (Pareto, 2014). The knowledge level reflects the tutee’s accumulated game-playing experience and what it learned so far from the tutor’s responses to asked questions. The robot starts at a novice stage, meaning it only makes random suggestions about which cards to play and it expresses uncertainty about what to do by asking the tutor for guidance. When the tutee gets more “knowledgeable,” it expresses more self-confidence, its suggestions improve, and the suggested card can be selected automatically if the choice is agreed upon by the tutor. This way, the game-playing behavior of the robot tutee changes over time, and the tutee appears to be learning. Furthermore, the robot is designed to be extroverted, positive, and cheerful concerning the players’ gameplay progression and about the tutor’s and its own performances.

**FIGURE 3:**
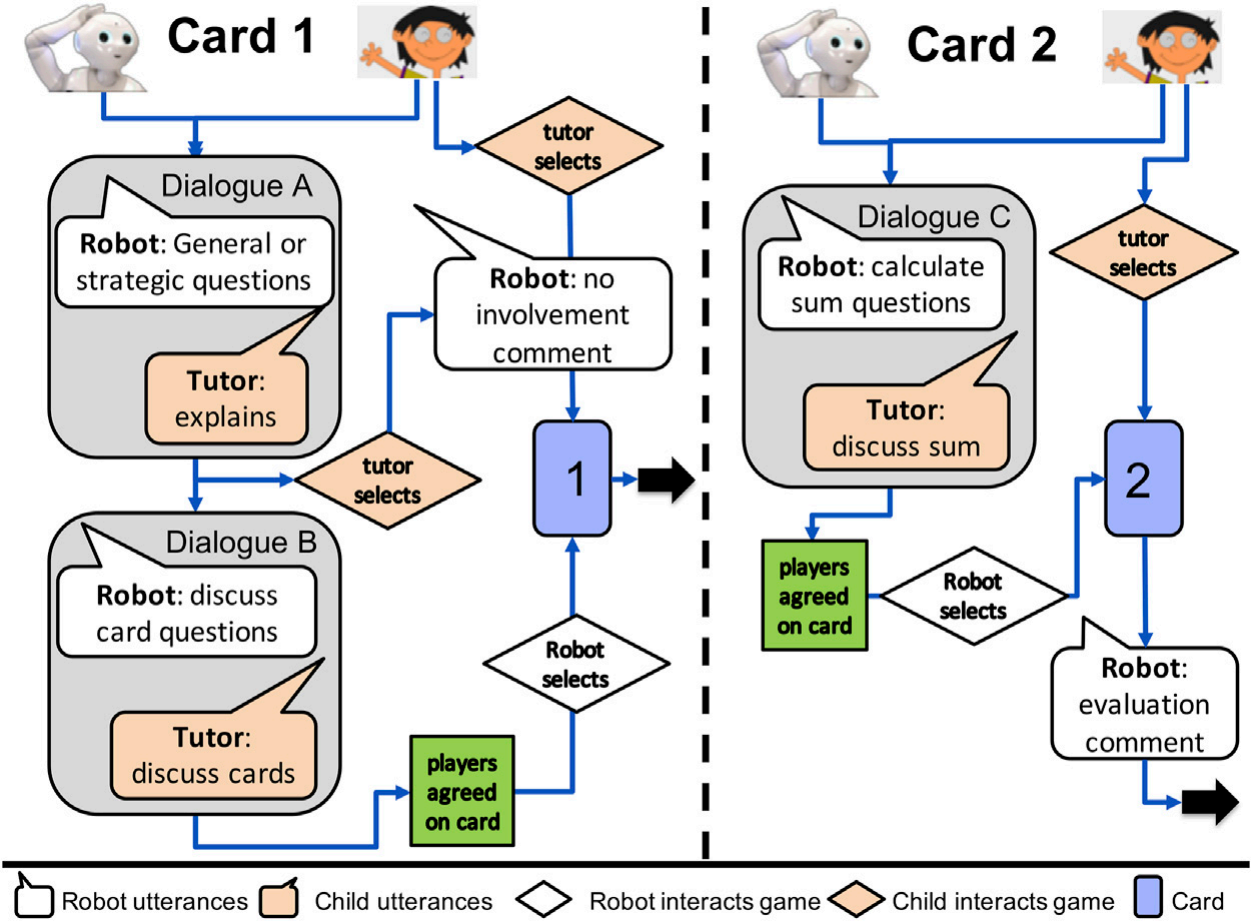
Interaction protocol of the START system, as a general action schema of the robot tutee.

The interaction protocol for the robot (see Figure 3) follows the structure of the mini-challenges: to discuss selection strategies, to discuss card alternatives and calculate sums, to select cards, and evaluate the result. The robot initiates four types of dialogs by asking 1) general questions about how to play the game, 2) strategic questions about how to find a matching pair cleverly, 3) computational questions involving arithmetic calculations of the proposed cards, and 4) conceptual questions about the base-10 system. The robot can initiate dialogs by asking questions or making comments several times during a mini-challenge, depending on the tutor’s actions and responses. The flowchart diagram in Figure 3 shows the general action schema of the robot tutee.

### 3.3 Research design

The study followed a within-subject design where participants interacted with both a robot tutee (RT) and a child tutee (CT) on different occasions the same week and 1 day apart, as in the study by Serholt et al. (2022). To avoid ordering effects, the conditions were counterbalanced for half of the participants.

### 3.4 Participants

The study was conducted in an empty classroom at a primary school in Sweden, comprising 20 students: 10 sixth graders (*N* = 10; 5 girls; 12–13-year-olds) and 10 third graders (*N* = 10; 6 girls; 9–10-year-olds). The sixth graders had previous experience both playing the graphical arithmetic game and interacting with the robot earlier in the START project (Pareto et al., 2019; Barendregt et al., 2020; Serholt et al., 2020). The third graders had no previous experience with the game. There were two brothers among the children who played together; the other couples were only familiar from the school.

### 3.5 Procedure

The children provided written assent and their legal guardians provided written informed consent before the study. Each session took 35 min and consisted of inviting the tutor (and CT when applicable) to the empty classroom where they were given a brief introduction to the study and asked to confirm their assent to be video recorded. Thereafter, the game and video cameras were started. For both conditions, the tutors’ mathematics teacher was responsible for the learning activity and provided guidance and support for the gameplay. Two researchers were present; one provided technical support for the START system when asked for by the participants or in case of system failures, while the other researcher handled the video equipment and data collection. If there was time left after the first game, the children were allowed to play again if they wanted to. Finally, debriefing sessions were held where the tutor responded to a set of questionnaires measuring their perceptions of the interactions (see Serholt et al., 2022).

### 3.6 Data collection and analysis

During the study, we captured the tutoring sessions and the children’s dialog through video recordings. There was one main camera capturing the entire scene with the players and game from behind (as in Figure 1) and one complementary camera next to the display capturing participants’ facial expressions and gestures not visible in the full-scene recordings. For this study, we conducted an in-depth analysis of six randomly selected tutors, of which three interacted with the robot first and three with the child first. These six tutors’ entire interaction sessions for both conditions were analyzed, amounting to 12 sessions in total. Data from game logs were used to determine actual game-playing duration and to compute achievement scores for each session.

The qualitative analysis tool MAXQDA^1^ was used for analysis. The tool supports not only qualitative data analysis of video recordings (Oswald, 2019) but also a mixed-method approach (Kuckartz, 2010). Combining frequency analysis with qualitative analysis conveys both the content by examples and the typicality of coded data (Erickson, 2012). Following such an approach, we combined a qualitative analysis of interaction dialogs with a frequency analysis of occurrences within themes. The frequency data were exported from MAXQDA and further analyzed statistically in SPSS^2^, version 27.

All videos were transcribed verbatim by the second author concerning verbal utterances according to the convention that each utterance continues until someone else starts talking or until there is a longer pause or change of topic. First, the video files were converted to sound files. Then the files were transcribed using the tool ScriptMe^3^ to document what was said by the involved actors. The transcriptions of the verbal dialog were double-checked and corrected manually before the analysis began. Then, the transcripts of the video sessions were imported into MAXQDA, where the transcripts were connected to the video clips. Three other types of events were added to the transcript: 1) game events, e.g., displays of new mini-challenges or scoring notifications, 2) interactions with the game, e.g., card selections by tutor or tutee, and 3) actors’ pointing gestures necessary to understand the verbal utterances, such as the pointing to specific cards accompanying verbal utterances “this and that.” The annotation schema was discussed among all authors, and the first author conducted all annotations. The first two categories, game events and player interactions, are distinct unambiguous events clearly visible on the game board. In category three, the pointing gestures were added only when the verbal transcripts included implicit references to visual objects on the game board. The final transcriptions comprise actors’ utterances and these annotated events.

The coding of the video transcripts proceeded in two phases. The first coding phase concerned connecting actions with actors and was conducted by the first and second authors. Five actors contribute to the tutoring dialog: the tutor and the tutee together with the game are the main actors. The game takes an active part in the tutoring task, as it communicates the mathematical challenge, provides feedback on the task, and is interacted with by the tutor and tutee. Important events from the game such as introducing a new challenge or scoring a mini-challenge and actors’ interactions with the game were also coded as actions. Additional actors in the tutoring scene were the teacher mentoring the tutoring sessions and a researcher handling technical issues with the game and the robot.

The second phase of coding concerned the instructional mechanisms in the tutoring session and was conducted by the first author. For this, the extended discourse structure framework IRFCE as presented by Duran & Monereo (2005) was used and modified to our specific context. The recommended two-step procedure was followed, i.e., to first identify segments of interactivity reflecting meaningful units of didactic sequences, and then interpret the segment according to the instructional mechanisms, herein action types, in the framework IRFCE. Each segment is coded with one action type described as follows. Due to the type of mini-challenge, all tutoring sessions followed a similar structure. The sessions started with an initiation (I) stating the problem, followed by a cooperative tutoring segment (C) discussing how to find the pair (sometimes omitted). Then, there was a cooperative segment discussing concrete card choices (C), followed by cooperative or solitary decisions of choosing cards, which correspond to a response (R) to the mini-challenge. Finally, the players provide feedback or evaluate their mutual accomplishments or each other (EF). The tutoring sessions typically followed the structure I(C)CREF, in which the first cooperative segment was either included or omitted.

**Initiation (I)**: the action-type “initiation” refers to when an actor comments or acts on a new mini-challenge, e.g., the tutor starts by rephrasing the displayed mini-challenge or the tutee starts with a question to the tutor.

**Cooperation—tutoring (C):** the action-type “tutoring” refers to segments where the relationship between the tutor and tutee is asymmetrical, i.e., a teacher–learner relation rather than an equal-partner relation. We have identified three typical situations: 1) *explain-to-tutee*: when the tutor takes initiative and guides or explains to the tutee, 2) *tutee-ask-question*: when the tutee initiates and asks the tutor a question seeking guidance and support, and 3) *ask-tutee-question*: when the tutor asks the tutee a teaching question, typically to check if the tutee knows something or is following the tutor’s reasoning.

**Cooperation—discuss card choice (C)**: the action type “discuss card choice” refers to segments where the tutor and tutee discuss which cards to choose before making their choices, typically a symmetrical and collaborative action type. The segment is considered collaborative if both players take part with at least one action each.

**Response—make card choice (R):** the response category in the IRFCE framework will, in our case, represent the response to the entire mini-challenge, to choose the cards. There are two card choices per mini-challenge, and each decision can be made in four different ways: 1) by the tutor alone, 2) by the tutee alone, 3) by being discussed between the tutor and tutee as described in the previous action type, or 4) by being discussed between the tutor and teacher only, i.e., excluding the tutee from the decision. The decision type is determined by the discussion preceding the card choice (or lack thereof).

**Evaluation/feedback (EF):** for the purpose of our analysis, we have chosen not to differentiate between evaluation and feedback as action types, since these actions are rather similar and mostly occur at the end of the mini-challenge. Feedback is also given by all actors, not only from the tutor to the tutee. Any comment on game progression, playing performance, or the other actor’s actions, and reflections on their discussion or previous response is coded as evaluation/feedback. After segment coding, the EF category was analyzed further and subdivided into categories according to whether the evaluation feedback concerned the gameplay, mathematics, the other player, or oneself.

Finally, the characteristics of the RT and CT tutoring sessions with respect to the IRFCE framework were analyzed by comparing group means between conditions of typical IRFCE action frequencies. The purpose was to examine characteristic differences between tutoring a robot and tutoring a child. We used the paired *t*-test method in SPSS to analyze group means.

## 4 Results

This section is organized as follows: first, we describe general information of the tutoring sessions in both conditions regarding the number of actions, number of completed mini-challenges, and correctness ratios of these mini-challenges, as a basis for the analysis of action frequencies. Then, we describe characteristic differences between the conditions that we have found, in the order a typical tutoring session proceeds, i.e., in the initiation, the tutoring segment, the discuss card segment, the response segment, and finally, the evaluation/feedback segment.

The results are based on the six tutors T1–T6 and the 12 tutoring sessions RT1–RT6 for the RT condition and CT1–CT6 for the CT condition. Segments in the video transcripts are denoted as *[tutoring session, id*_*pos*_ *]* where *id*_*pos*_ is the position identifier in the video transcript generated by MAXQDA.

**FIGURE 4:**
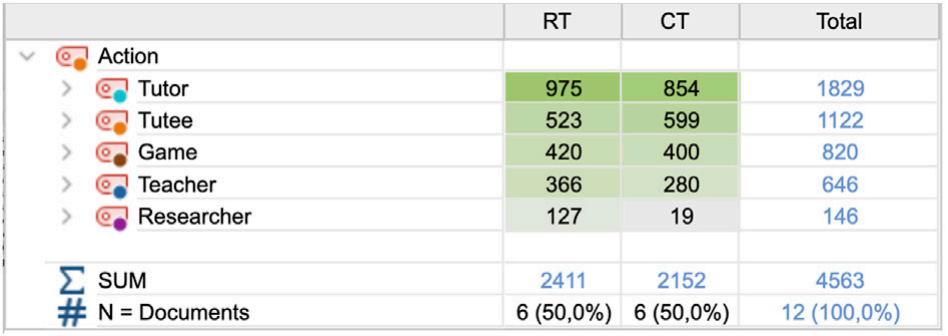
Number of actions distributed between conditions and actors.

The total number of coded actions for all actors in these sessions was 4,563 distributed between actors as shown in Figure 4. The level of activity is similar in the two conditions, with the tutee being slightly more active in the CT condition than in the RT condition, while on the contrary, the tutor is more active in the RT condition than in the CT condition. Mentoring actions by the teacher are more frequent in the RT condition, as are technical support actions provided by the researcher since the robot required more technical assistance than the game alone. There were sequences in the RT condition when the robot stopped responding, mainly in the first session. These sequences constitute 2% of the transcripts and were excluded from analysis.

There was 35 min allocated to each session, including the startup time when the teacher introduced the task. Active game-playing time varied between 20 and 33 min. A mini-challenge is chosen as the unit of analysis; there were 104 mini-challenges played in each condition, in total, 208 mini-challenges and 329 min of analyzed and coded video recordings. It is a coincidence that the total number of mini-challenges in both conditions is the same since the players set the path of solving the mini-challenges within the allocated time. A paired-samples *t*-test was conducted to compare the number of mini-challenges in the RT and CT conditions. There was no significant difference in the number of mini-challenges for RT (M = 17.33; SD = 5.428) and CT (M = 17.33; SD = 4.082) conditions; t (5) = 0.000 and *p* = 1.000, meaning that there is no statistical difference between the conditions regarding the number of mini-challenges the players accomplished during the tutoring sessions. The tutors managed to play well in both conditions and solved about 90% of the challenges. A paired-samples *t*-test was conducted to compare the correctness ratio in the RT and CT conditions, and there was no significant difference concerning how well they managed to play (i.e., correctness ratio) in the RT (M = 93.83; SD = 15.105) and CT (M = 90.67; SD = 10.033) conditions, t (5) = 0.856 and *p* = 0.431. These results indicate that the tutee type (child or robot) does not affect the players’ performance in solving mini-challenges. Hence, we can dismiss playing performance as a possible explanation for other differences we found in the two conditions.

### 4.1 Between-condition differences in initation (I)

**FIGURE 5:**
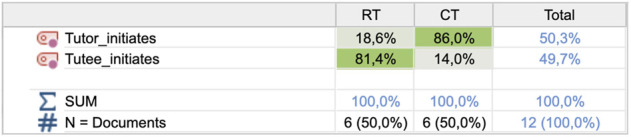
Frequencies of initiation actions by the tutor and tutee, respectively, for the two conditions.

One observed difference concerned who was taking the initiative to start the dialog after the game displayed a new mini-challenge to the players. The child tutor initiated the dialog in 86% of the challenges in the CT condition, compared to 18.6% in the RT condition as shown in Figure 5. All tutor initiations in the CT conditions were variations of restating the challenge such as “*we must find the sum of X*.” In the RT condition, the tutor either restated the mini-challenge or made a general statement like “*one more round*” or “*let’s do one more*.” This result shows that the tutors were more passive toward the robot tutee even when it was silent and rarely restated the challenge for the robot tutee as they did with their human collaborators. A paired-samples *t*-test was conducted to compare tutor initiations. There was a significant difference between the number of tutor initiations in the RT (M = 3.00, SD = 3.225) and CT (M = 12.33; SD = 4.803) conditions; t (5) = -5.470; *p* = 0.003. This result suggests that the tutor is more likely to start the dialog when tutoring the child than when tutoring the robot.

Regarding tutee initiations, the situation was the opposite. The tutee initiated the dialog in 81.6% of the mini-challenges in the RT condition and 14% in the CT condition (see Figure 5). Of the 79 robot initiations, 60 were direct questions prompting the tutor to explain or guide (see Section 4.3 for examples). In the remaining initiations, the robot tutee made comments on the task being challenging, e.g., “*This is a bit difficult, I think*” or “*now it seems more difficult to find the sum*.” There was also a significant difference between tutee initiations in the RT conditions (M = 13.17, SD = 3.817) and CT conditions (M = 2.00; SD = 2.757); t (5) = 4.793; *p* = 0.005, indicating that the robot tutee is more likely to initiate the dialog than the child tutees.

### 4.2 Between-condition differences in cooperation–tutoring (C)—tutee questions

**FIGURE 6:**
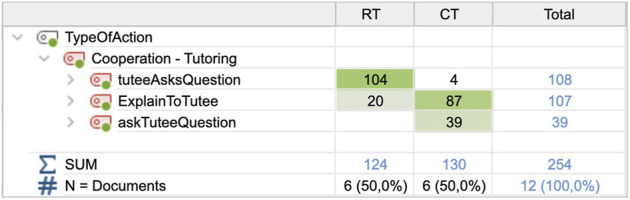
Frequencies and types of tutoring actions for the two conditions.

Another observed difference in the tutoring sessions concerned the cooperative tutoring segments. There were two common situations: either the tutee triggered an explanation from the tutor by asking a question or the tutor spontaneously explained to the tutee, and these are examined further as follows. A less common type of tutoring was teaching questions to the tutee, such as “*if we take 20 here plus 7, what is the sum?*” The frequencies and types of tutoring segments in the respective conditions are presented in Figure 6.

**FIGURE 7:**
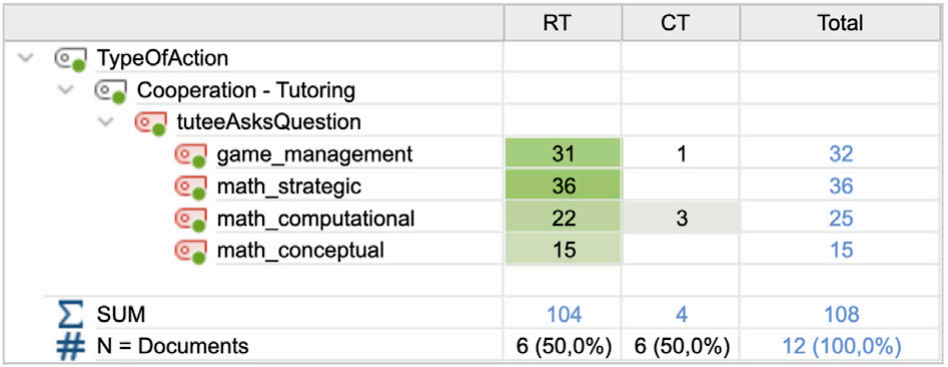
Frequencies and types of tutee questions for the two conditions.

In the CT condition, the tutee only asked four questions in total between the six tutees. Being of similar types as the robot tutee questions (see Section 3.2), all tutee questions were organized into these categories (see Figure 7). In the RT condition, the tutee asked the tutors questions 104 times during the sessions. The robot questions were activated according to the interaction protocol of the START system (see Figure 3). There was a significant difference between the number of tutee questions in the RT (M = 17.00, SD = 3.347) and CT (M = 0.67; SD = 0.516) conditions; t (5) = 13.003; *p* = 0.000.

Some examples from the dialogs are provided to illustrate the nature of the tutee questions. In the CT condition, one tutee asked if only the taken cards were replaced in the next round (i.e., game management) and three questions were about the sum to be searched for, such as [*RT6, 100*]: 

**CT:** What did you say we should add to?

**Tutor:** 35.

**CT:** Aha.

From the RT condition, we provide some tutee questions in each category that can stimulate the tutor’s learning in different ways. In the first example from game management questions, the tutee prompts the tutor to explain what the mathematical challenge is about [*RT6, 27*]:

**RT:** Tell me, what should we do?

**Tutor:** It says here how much, the sum, and we have numbers on each side. We should find which cards.

**RT:** Fine. Then I know.

In the second example, the tutee asks a question about all mini-challenges, i.e., a generalization from instances, of which the tutor is unsure and seeks confirmation from the teacher [*RT3, 115*]:

**RT:** Is there always a pair that matches the sum?

**Tutor:** Yes. There is always a pair that adds to the sum. It must do that, doesn’t it? [The tutor seeks help from the teacher.]

**Teacher:** Yes, it does.

Strategic questions concern different methods to find the pair. The first example prompts the tutor to explain a general method of how to find pairs [*RT5, 257*]:

**RT:** This is a bit difficult. How do you search for cards that fit with the sum?

**Tutor:** I go through the cards and see which fits.

**RT:** Yes, I know that. But how do you add the numbers?

**Tutor:** I go through the cards and see if they fit and start with the ones. If they add to 2 or 12.

While in the second example, the tutee prompts a generalized idea to the tutor by seeking confirmation of a suggestion [*RT6, 290*]:

**RT:** How should we think to find the pair? Check if both the red and the orange blocks match?

**Tutor:** Add the cards together.

Computational questions are about the mini-challenge sum and how to form the addition. In the following example, the tutee prompts the tutor to reveal the solution and it shows how the robot simulates contextual awareness by referring to visual information on the game board (i.e., the sum) [*RT4, 261*]:

**RT:** Should we find two cards that are 98 together?

**Tutor:** Yes, we should.

**RT**: I thought so too. Which two cards should we play to get 98?

**Tutor**: Yes, 50 and 48.

Conceptual questions concern relations between the graphical game representation and the ordinary symbolic representation or structural properties of these representations as in the following example, where the tutee refers to a structural observation and ask the tutor for an explanation [*RT3, 357*]:

**RT:** There is something I do not understand. Why are the red and orange blocks in different compartments on the game board?

**Tutor:** Because one side is the ones, the other the tens.

**RT:** You teach me greatly!

### 4.3 Between-condition differences in cooperation–tutoring (C)—tutor explanations

**FIGURE 8:**
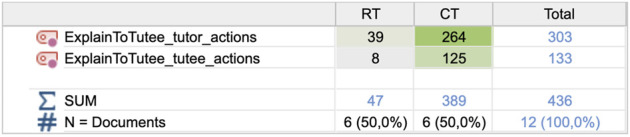
Frequencies of actions in explanation segments by the tutor and tutee**,** for the two conditions.

The second most common tutoring segment after tutee questions is spontaneous explanations by the tutor (see Figure 6), which deserves further investigation. Explanations are four times as common in the CT compared to the RT condition, but the characteristic difference regarding explanations between the conditions is more profound than that, due to similar differences in the number of actions in each segment and the number of words in each action. Therefore, we explored the number of tutor and tutee actions within these explanation segments, as shown in Figure 8. Paired t-tests revealed a significant difference between number of tutor actions in the explanation segments in the RT (M = 5.67, SD = 5.989) and CT (M = 43.83; SD = 31.057) conditions; t (5) = −2.871; *p* = 0.035. Likewise, regarding the number of tutee responses as reactions to the tutors’ explanatory actions, there was also a significant difference between the RT (M = 1.00; SD = 1.265) and CT (M = 22.33; SD = 19.211) conditions; t (5) = −2.778; *p* = 0.039.

Moreover, the number of words in the actions also differed between conditions. In the CT condition, the 264 tutor explanation actions comprised 4,456 words, i.e., 17 words on average per statement. The most common word “we” (266 times) indicates an inclusive intention of the tutors, followed by “this/that” (242 times) frequently used together with pointing at the game board. Tutee responses to tutor explanations were in general very short, on average 2.1 words per response. The most frequent response was a mumbling “mmm” (about 50%), followed by “yes” or a number. The following examples illustrate how explanations can unfold in the CT condition using different tutoring approaches [*CT1, 134*]:

**Tutor:** Then, we should find about 30 [the sought sum is 69].

**Tutor:** Then, we see here… 8 plus 6. So 8 plus 2 is 10 and 10 plus 6 is… Or 10 plus 5, that is 15, so the two at the top do not work. It must be below 60. 30 + 30 is 60. Then 8 plus 1 is…

**CT:** 69.

**Tutor:** Yes, you can take them. These two. You must start to the left. [Tutor points at the two cards.]

As compared to [*CT6, 92*]:

**Tutor:** Yes, we can think so, what do they fit with? If we choose to test with 27.

Is there any card here that would fit with 27? So, if that is 35 and this 27.

**CT:** Mmm.

**Tutor:** Then, we need 8 to get 35. But we do not have that. So, we can discard the first, it does not fit.

**CT:** Mmm.

**Tutor:** The next is 4. If we… What is 35 minus 4?

In the RT condition, four out of six tutors provided in total 39 unprompted explanations to the tutee. The explanations to the robot were of the same character as explanations to the child, and the eight responses from the robot were all general statements such as [*RT4, 54*]:

**Tutor:** Here, we see that you have… you have an 8. Or 8. And here you have 38. There you have 49 and 11. And one of your cards should fit with my cards.

**Tutor:** Pepper?

**RT:** Yes, I am here.

**Tutor:** Okay, good that you are following.

**RT:** I promise to listen better.

### 4.4 Between-condition differences in cooperation–discuss card choice (C) and response (R)

**FIGURE 9:**
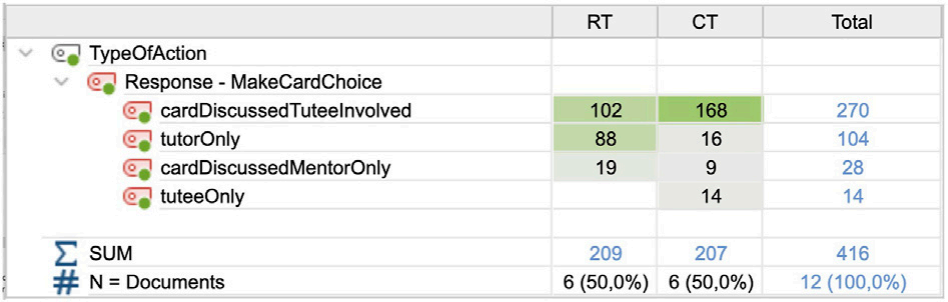
Frequencies and types of player collaborations in card selections for the two conditions.

A fourth difference between the conditions concerned how much the tutors involved the tutees in decisions. In the CT condition, the tutee was either involved in or made the decision alone in 88% of all decisions made (168 + 14 of 207 in Figure 9) compared to less than 50% (102 of 209) in the RT condition. There was a significant difference between how much tutees were involved in decision-making in the RT (M = 17.00; SD = 6.870) and CT (M = 30.33; SD = 6.186) conditions; t (5) = −2.810; *p* = 0.038.

These tutee-involved discussions selecting cards also differed in characteristics between conditions. In the RT condition, the robot tutee most often initiated the card discussion with one of its pre-programmed questions. The RT referred to cards with their number representation and sometimes evaluated the second card choice by performing the addition as a question to the tutor or as a statement (example 1 as follows). The RT made its suggestions when its knowledge level was higher but asked the tutor for confirmation and withdrew if the suggestion was not confirmed (example 2) or rejected (example 3). This meant that the tutor was in charge of all choices.

RT condition, example 1*,* second card tutee’s turn, sum 39, they already played tutor’s card 13 [*RT1, 341*]:

**RT:** Which of my cards 42, 26, 23, or 24 is best, do you think?

**Tutor:** 26.

**RT:** Yes, 26 plus 13 is 39. I will take that!

RT condition, example 2, first card tutee’s turn, RT suggests own card [*RT3, 473*]:

**RT:** Do you think my 37 is a good choice?

**Tutor:** Uhhh…

**RT:** Okay, maybe I was wrong. Which card should we choose?

**Teacher:** Anyone who knows?

**Tutor:** 37, that is the same card…

**RT:** I will take card 37.

**Tutor:** Good.

RT condition, example 3, first card tutor’s turn, RT guesses one of the tutor’s cards [*RT4, 106*]:

**RT:** If you take your 46, can we get 52 then with my cards? 

**Tutor:** No.

**RT:** You know better. Which card will you choose?

**Tutor:** Can we think a bit?

**RT:** Now I am thinking.

**Tutor:** Okay, fine.

In the CT condition, the card choosing discussion varied more between sessions, as expected when two humans collaborate. The players made extensive use of demonstratives such as this and that, accompanied by gestures referring to game board visuals. Their discussions were most often initiated by the tutor, but the tutors had different teaching styles. The first two are tutor-led. In example 1, the tutor reasoned aloud to walk the tutee through the proposed calculation of the sum all at once, whereas in example 2, the tutor posed questions to ascertain the tutee was following. CT condition, example 1, sum 93 [*CT4, 157*]:

**Tutor:** Since it should be 93… maybe we should take something with four of these. That we have here, four tens. Or perhaps… But it only works with 4. We must have 4 on each side, and you have one there. We add this then, and that over there. Then it becomes… First 80, then it is 9 plus 5 [looking at the ones] and that is…

**CT:** 93.

**Tutor:** What did you say?

**CT:** 93.

**Tutor:** 9 plus 5.

**Teacher:** It is 8 on that side. [Corrected the tutor who misread a card.]

**Tutor:** Yes, of course, 8 plus 5.

CT condition, example 2:

**Tutor**: And here, we have 14 on your side. [*CT6, 197*]

**CT:** Mmm.

**Tutor:** And now, we need 27 on my side. Can you see one? It has seven red and two orange [blocks].

**CT:** Yes.

**Tutor:** Yes, where?

**CT:** Here. [Tutee points at one of the tutor’s cards.]

**Tutor:** Yes, you can click on it. And I take the other.

In the next two examples, the tutors encouraged the tutees to be actively involved in the decisions and simultaneously scaffolded them. In example 3, the tutor suggested the first card and then asked the tutee to make the second choice, whereas in example 4, the tutor limited the choices and told the tutee to focus on four cards only. In these examples, both tutees sought acceptance from their tutors before they took an action, but there were cases when the CT made choices on their own. CT condition, example 3: [*CT2, 276*]:

**Tutor:** 61. I think that… [the tutor points at a card].

**CT:** That? [The tutee points to the same card.]

**Tutor:** Yes, and…

[Quiet for 20 seconds.]

**Tutor:** What do you think?

**CT:** I think this and then that… since they become... [Tutee points at the cards.]

**Tutor:** Yes, that adds to the number.

**CT:** Yes, then we take that.

CT condition, example 4: [*CT1, 152*]:

**Tutor:** So, the two at the top there and the two at the top there. Do you want to try it on your own?

**CT:** This and that. [Tutee points at two cards that he has chosen.]

**Tutor:** [Tutor nods.] You must start to the left.

The second most common situations were tutor-alone decisions, occurring 16 times with the CT (M = 2.67; SD = 4.179) *versus* 88 times with the RT (M = 14.67; SD = 8.824); the difference was significant t (5) = 3.065; *p* = 0.028. Hence, the tutor was not as likely to involve the RT in choosing cards compared to the CT. There were some typical situations when the RT was ignored: a few times the robot got into a passive mode and kept quiet, but more often the tutor ignored the RT when appearing to be engaged in the gameplay or thinking about the problem. In the CT condition, the tutor sometimes pointed and gave the tutee directive to play a particular card, which we did not consider constituted involvement in the decision. Interestingly, 15 of the 16 occasions when the CT was ignored were from the tutor group who played with the RT first. It is thus possible that the order of the sessions affected the tutors’ behaviors such that the behavior of ignoring the robot tutee was transferred to the child tutee session.

### 4.5 Between-group differences in evaluation/feedback—tutee and tutor feedback

**FIGURE 10:**
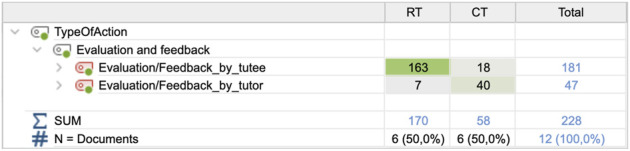
Frequencies of evaluation feedback by the tutor and tutee**,** for the two conditions.

The last identified difference between conditions concerned the amount of evaluation feedback that was given by the actors, in particular, feedback provided by the tutee. In the original IRCFE framework, only tutor feedback to the tutee is considered, but here we include all actors’ evaluation feedback. In the CT condition, the tutees gave their tutors feedback 18 times (M = 3.00; SD = 3.688), compared to 163 times (M = 27.17, SD = 7.627) in the RT condition (Figure 10). The difference was significant; t (5) = 8.425; *p* = 0.000.

There were five types of feedback, categorized according to feedback content (gameplay or mathematical) that target their joint activity, or feedback directed to the other player or oneself:

*1) Gameplay:* statements concerning the progression in the game
*2) Mathematical:* a mathematical evaluation, here, calculating if the proposed cards sum up to the correct value
*3) Acknowledge other*: statements where either the tutee acknowledges the tutor or the tutor acknowledges the tutee, by showing appreciation of the play partner’s accomplishment in some way
*4) Self-reflection:* evaluations concerning own accomplishments (either positive or critical)
*5) Questioning other:* statements questioning what the other playmate is proposing or suggesting

**FIGURE 11:**
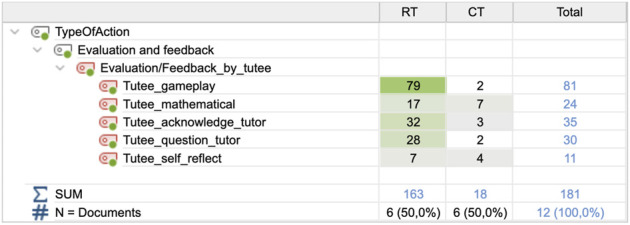
Frequencies and types of evaluation feedback by the tutee, for the two conditions.

The distribution of feedback types for the tutee feedback is shown in Figure 11.

In the RT condition, the gameplay feedback was about playing performance and scoring, like “*Great, our first point*” or “*Look, we have 7 points now*.” Mathematical feedback was approval followed by control calculations, such as “*Good choice. 47 plus 17 equals 64*.” The RT acknowledged the tutor 32 times compared to 3 in the CT condition. The types of acknowledgments were “*You are a great teacher*,” “*I learn a lot from you,*” “*You know everything!*” and “*You are the best*.” The CT acknowledgments to the tutors were similar but more neutral: “*Nice work*” and “*Good*.” The RT made self-reflective comments such as “*I’m good at this, right?*” and “*I was right, I’m so happy*,” or worries such as “*I find this difficult*.” The RT also questioned the tutor more than the CT, 28 times compared to twice. Such questioning seemed to stimulate the tutor to reflect on their proposed actions and re-evaluatge their thinking, e.g., example 1 when the robot’s questioning was correct [*RT5, 164*]:

**RT:** But, do 19 and 18 add to the sum of 33?

**Tutor:** No… it does not.

**Tutor:** No, it does not. My thinking is wrong.

Example 2 When the robot questioning was incorrect [*RT6, 147*]:

**RT:** But, is 5 plus 10 really 15?

Example 3 When the robot questioning is not supported with a reason [*RT2, 598*]:

**RT:** Oops. Did we think correctly now? Maybe we need to think more before we choose the first card.

**Tutor:** I think we were right.

It was interesting to note that only the CT who knew the tutor well expressed self-reflective comments and questioned the tutor; an explanation can be that this tutee felt secure enough to oppose and give feedback to the tutor, contrary to the other tutees. Regarding the tutors’ feedback and evaluation of their tutee, they were more inclined to comment and verbally acknowledge the CT than the RT, and they also commented on the progression more in the CT condition.

## 5 Discussion

Even though learning-by-teaching has shown evidence to be effective for tutor learning for decades (Duran, 2017), such learning effects are contingent on the nature and the quality of the tutor–tutee interactions (Roscoe and Chi, 2007). Since the quality of the tutoring activities is crucial, learning effects may not transfer from one situation to another nor from one tutoring couple to another (Topping, 2000). Rather, potential learning gains depend on the behaviors of the tutors and the tutees. In general, the learning-by-teaching approach provides opportunities for the tutor to learn from the following activities (Duran, 2017; Koh et al., 2018): 1) preparation that involves reviewing and organizing material, possibly extending the knowledge base if considered necessary; 2) explanation which means to verbalize and try to make sense of current knowledge; 3) answer tutee questions, which requires an understanding of the question, recalling of appropriate knowledge, and the ability to contextualize and formulate an answer; 4) reflect on others’ ideas or ways of thinking and assimilate this with current understandings; and 5) be questioned or face tutee confusion, possibly leading to reflection and extension on current understanding. All five activities have, independently, the potential to result in tutor learning. However, tutor learning gains require that the tutor engages in reflective knowledge building (Koh et al., 2018), but it also depends on the tutee’s ability and willingness to engage in asking relevant questions, propose their own ideas or areas of confusion, and provide feedback to the tutor so the tutoring can be evaluated (Duran, 2017).

In our study, the tutors were not asked to do any preparation for the tutoring session in advance, except that they had previously played another mini-game and they knew that their task was to teach the robot and a younger child to play the game. They were accustomed to the graphical representation and the general game features, even though the game they played earlier was mathematically easier. Hence, there were no explicit preparation activities before the tutoring sessions, which may be omitted when using games (Topping et al., 2003). Explanations, on the other hand, were extensively used in the tutoring sessions with the younger child tutees, the CT condition, but not with the robot tutee. A few tutors attempted to give the robot tutee explanations similar to the child tutee, but gave up long explanations in favor of shorter keyword-based statements, due to the robot’s inability to interpret long explanations. On the contrary, all tutors except one gave long elaborated explanations to their child tutees. Thus, the CT condition provided ample opportunities for tutors to develop their understanding when having to verbalize their thoughts (Topping et al., 2003) and self-explain (Roscoe & Chi, 2007; Koh et al., 2018) in order to formulate comprehensive explanations to their tutees who were all attentive listeners. Similar opportunities to learn from explanations did not arise in the RT condition. This was likely due to the tutors’ knowledge of the technical limitations of robots and their speech recognition technology in interpreting verbal communication based, in part, on their earlier experience of the robot in question (cf. Serholt et al., 2020). Also, since the tutoring activity involved mathematics in a non-traditional way (using the graphical representation), it seems reasonable to assume that the tutors were engaged in knowledge-building activities, rather than simple knowledge telling yielding no tutor learning (Duran, 2017), when they explained to their tutees.

Regarding tutee questions, the situation was reversed. The child tutees asked almost no questions at all. This is not surprising, since most students are not frequent question askers (Graesser and Person, 1994). Also, the younger child tutees may have felt too insecure in the tutoring situation with a three-year-older tutor to ask questions. That the only tutee asking questions knew the tutor well beforehand is an indication supporting that idea. The robot tutee, on the other hand, posed plenty of mathematical questions of different kinds to the tutors, questions that were often challenging for the tutor to answer. These questions are part of the robot tutee design. An advantage of such externally constructed questions is that the tutee can challenge the tutor with deeper, more explanatory questions than the tutor self or a child tutee can be expected to ask spontaneously. The tutee questions and the tutee questioning concerned the mini-challenge in the game and were directly related to the game scenario or the tutor’s actions in the game. The robot’s limitations concerning speech recognition became less apparent when the robot asked questions since the action’s relevance depended on the game situation, and responses can be anticipated as opposed to freely formulated explanations, for example. Martino & Maher (1999) showed that posing timely and challenging questions can stimulate mathematical learning and is one explication behind tutoring effectiveness. It is not enough, however, to expose learners to good questions, they must also engage with the inquiry. In the learning-by-teaching situation, engagement comes from the so-called protégé-effect (Chase et al., 2009). Duran (2017) argued that the learning-by-teaching literature review shows evidence of tutee questions being effective and stimulating collaboration toward tutor–tutee joint understanding. Nevertheless, such reflective power of questions depends on the quality of the question. The robot tutee questions are all constructed as a means to stimulate tutor reflections of strategic or computational nature or reflections on important arithmetic concepts such as number structures. A previous version of the tutoring game used in our study that deployed learning-by-teaching with a teachable agent instead of the robot tutee has shown to be effective for tutors’ mathematical learning (Pareto, 2014). Since the robot questions are generated from the same schema, it is plausible that the robot tutee questions provide ample opportunities for the tutor to learn from tutee questions in the RT condition. In the CT condition, there were very few such opportunities.

The tutoring context is also of importance for the tutor’s learning. Several researchers have pointed out the importance of a well-defined and well-structured tutoring task (Topping, 2000; Belpaeme et al., 2018; Jamet et al., 2018) and argue that games are suitable candidates for the tutoring task (Topping et al., 2003; Duran & Monereo, 2005). The game-playing activity with visual display functions as a boundary object (Star & Griesemer, 1989) for the robot–tutor collaboration and communication, which focuses the attention on a joint activity and naturally restricts the communication space. Also, most games have structured rules and well-defined goals. Robot-augmented games like ours that supply the robot with contextual information provide more context-aware robot tutees than robots with only built-in features, e.g., the robot can “see” what is displayed on the game board and “knows” what the tutor has previously done in the game. However, a visual game display as a boundary object for the activity also introduces the possibility to use gestures and implicit language with demonstratives, which may counteract a basic learning objective to use mathematical language. In the RT condition, explicit mathematical language was needed to make the robot tutee understand, whereas in the CT condition, the players frequently utilized demonstratives and gestures. It was also evident that the child tutee and the tutor, being peers of similar age who shared vocabulary and linguistic styles (Duran & Monereo, 2005), made the dialogs in the explanations and in card selection discussions fluent, as opposed to the dialogs with the robot. This finding was also supported by the self-reported questionnaires regarding the ease of collaboration and communication in favor of the child tutee condition (Serholt et al., 2022). Regarding enjoyment and willingness to play again, there was no significant difference between the two conditions. Being a less capable tutee rather than a peer or tutor robot may have affected the tutors’ tolerance toward the technical limitations of the robot and their perception of enjoyment (cf. Chen et al., 2020).

Finally, from participating in the game sessions and from observing the video recordings, we have observed that all tutors in our study had their style of tutoring, particularly in the CT condition. For example, one tutor provided long intricate explanations while another provided no explanations but focused only on the card choice discussion, whereas yet another tutor treated the child tutee more as an equal partner and discussed everything with the tutee. In the robot condition, on the other hand, the tutoring styles were more uniform since the robot tutee was the initiator and driver of many dialogs and the discussions were, thus, both influenced by the robot design and restrained by the robot’s limitations to understand explanations and reasoning.

### 5.1 Limitations and future work

Limitations to this study include few participants, few within-subject comparisons, and studying a tutoring activity involving one type of mathematical mini-challenges only. Future work includes further analysis of the different teaching styles between participants, which seem to vary in particular for the child tutee condition. Also, we only investigated learning opportunities for the two conditions; actual learning-by-teaching effects need to be studied as well.

## 6 Conclusion

We have explored how a learning-by-teaching situation using a mathematics game with a robot tutee compares to an equivalent situation with a younger child tutee, concerning frequencies and characteristics of the tutoring activities, teaching styles, and learning opportunities for the two conditions. We found that the tutoring situations differed significantly between the robot tutee and the child tutee conditions in the following dimensions, regarding frequency and characteristics:

1. The robot tutee takes significantly more initiative compared to the child tutee, and it often drives the dialog. In the child tutee condition, the tutor is the main initiator.
2. The robot tutee asks significantly more questions than the child tutees. The questions often challenge the tutor and can prompt the tutor with new ideas.
3. The child tutees receive significantly more explanations from the tutor than the robot tutee, and these explanations are also substantially longer, more elaborated, and make more use of gestures and implicit language.
4. The tutors involve the child tutees significantly more in the task’s decisions than the robot tutee. The robot tutee is more often ignored.
5. The robot tutee provides significantly more evaluation feedback to the tutor than the child tutees. The feedback mainly concerns gameplay, mathematics, acknowledgments to the tutor, or questioning the tutor’s proposals.

There were individual variations between tutors in the child tutee condition. In contrast, the robot tutee condition was rather uniform since the dialog was more robot-driven.

Based on the aforementioned significant differences in tutoring in the two conditions related to the theory and pedagogical underpinnings of the learning-by-teaching approach, we have identified the following learning opportunities for the tutor in the two scenarios:

**Table:** 

**Child tutee condition**
Benefits	1. Opportunities to engage in explanations to the tutee (Roscoe and Chi, 2007; Koh et al., 2018)
	2. Smooth dialog and collaboration (Duran & Monereo, 2005)
	3. Social responsibility for a younger child creates motivation (Chase et al., 2009)
Drawbacks	1. Little input from child tutees
	2. Gestures and implicit language instead of mathematical language
**Robot tutee condition**
Benefits	1. Opportunities to answer the robot tutee’s questions (King 1994; Martino & Maher, 1999; Duran, 2017)
	2. Plenty of feedback from the robot tutee (Roscoe & Chi, 2007; Duran, 2017)
	3. Use of mathematical language (Topping et al., 2003)
Drawbacks	1. Limited opportunities to practice explanations
	2. Limited conversational freedom in the dialog (pre-programmed keywords and phrases)

To conclude, the two learning-by-teaching situations with a robot tutee and a child tutee both provide good learning opportunities for a tutor, but in different ways.

## Data Availability

The transcripts supporting the conclusion of this article will be made available by the authors, without undue reservation.

## Footnotes

1
https://www.maxqda.com/.

2
https://www.ibm.com/products/spss-statistics.

3
https://www.scriptme.io/.
